# Evaluation of the aesthetics and clinical findings in patients with missing maxillary lateral incisors treated with a 10-year interval

**DOI:** 10.1093/ejo/cjae018

**Published:** 2024-04-24

**Authors:** Cecilia Hedmo, Rune Lindsten, Eva Josefsson

**Affiliations:** School of Health and Welfare, Jönköping University, Jönköping, Sweden; Department of Orthodontics, The Institute for Postgraduate Dental Education, Jönköping, Sweden; School of Health and Welfare, Jönköping University, Jönköping, Sweden; Department of Orthodontics, The Institute for Postgraduate Dental Education, Jönköping, Sweden; School of Health and Welfare, Jönköping University, Jönköping, Sweden; Department of Orthodontics, The Institute for Postgraduate Dental Education, Jönköping, Sweden

**Keywords:** aplasia, lateral incisors, clinical findings, orthodontics, Osseo-integrated implants

## Abstract

**Introduction:**

The most common treatment approaches for patients missing maxillary lateral incisors are implant replacement (IT) and orthodontic space closure (SC). Treatment techniques change and improve over time, and it is of interest to know if improvements differ between the methods.

**Aim:**

To compare the aesthetic outcome and other clinical findings in patients with one or two missing maxillary lateral incisors who were treated with a 10-year difference in time, with either orthodontic space closure or implant replacement.

**Material and methods:**

A total of 88 patients were included in the study. Forty-four patients treated between 2011 and 2018 were included as the latter cohort (LC). The LC was compared to the early cohort (EC; *n* = 44), treated between 2001 and 2008. A total of 132 teeth was analysed: 62 teeth in the EC (28 teeth in IT cases and 34 teeth in SC cases) and 70 teeth in the LC (34 teeth in IT cases and 36 teeth in SC cases). Long-term clinical and aesthetic outcomes were evaluated.

**Results:**

An improvement over time was found in crown length, BoP, papilla, the inclination of incisors, and overall appearance in IT cases and in crown colour and overbite in SC cases. A deterioration over time was found in crown length and BoP among the SC cases.

**Conclusion:**

Among the IT cases, an improvement in outcomes was noted over time. When comparing SC cases the colour of the crown and overbite had improved, while crown length and BoP had deteriorated over time.

## Introduction

Tooth agenesis, or a congenitally missing tooth or teeth, is a common disorder that carries significant functional, psychosocial, and financial burdens [1, 2]. The prevalence of missing maxillary lateral incisors is reported to vary between 1.5 and 2% [3, 4], making it a common feature in the field of orthodontics [5].

Treatment alternatives in patients missing maxillary lateral incisors are orthodontic space closure, prosthodontic rehabilitation, or autotransplantation. The most used are orthodontic space closure and orthodontic space opening to facilitate prosthetic replacement of the missing tooth or teeth [6–8]. When a missing lateral incisor is to be prosthetically substituted, this can be done using resin-bonded fixed dental prostheses, conventional fixed dental prostheses, or osseo-integrated implant-supported crowns [9, 10]. The most commonly used approach to prosthetically replacing a missing maxillary lateral incisor, and according to some of the best therapy [11], is osseo-integrated implant-supported crowns [7, 12, 13]. The choice of treatment approach depends on the age of the patient, available space in the dentition, status of adjacent teeth, type of occlusion, facial profile lip line, and contour of the gingiva [14–16]. However, in many patients, both treatment alternatives are considered equally good.

There are some known complications associated with implant-supported prosthetic replacement of maxillary lateral incisors, namely degeneration of soft tissue, discoloured gingiva, visible implants, and infra-occlusion of the implant suported crown [17–19]. Despite these unwanted complications, implant-supported crowns are considered to provide satisfactory long-term functional and aesthetic outcomes [12, 20–22]. Space closure, i.e. canine substitution, also face aesthetical difficulties such as deviating tooth colour, size, and form [23].

Both treatment techniques are well studied. The aesthetic outcome has been found to be equal even though there are some parameters that are in favour of space closure. Space closure should be recommended when possible [24]. Treatment techniques change and improve over time; however, it is of interest to know if improvements have differed between the methods.

The aim of the present study was to compare the long-term outcomes of treatments for agenesis of the lateral maxillary incisors over a time span of 10 years to determine whether treatment with implant therapy and space closure evolved equally over time.

The null hypothesis was that there was no improvement in aesthetic outcomes nor clinical findings over time for both treatment alternatives.

## Material and methods

This study comprised an early cohort (EC) treated between 2001 and 2008 and a late cohort (LC) treated between 2011 and 2018. These cohorts included patients with agenesis of one or two maxillary lateral incisors treated with either implant therapy (IT) or space closure (SC). Comparisons within the treatment alternatives were made over time. Hence, cases treated with SC in the LC were compared to SC cases in the EC. The same comparisons was made between the IT cases.

The Swedish Ethical Review Authority approved this study, diary number 2020-00301. All participants in this study were given written and oral information about the study and gave their written consent to participate.

A cross-sectional, retrospective study design was used. The subjects were recruited at the Institute for Postgraduate Dental Education, Jönköping, Sweden. All patients who had undergone implant therapy due to agenesis of one or two maxillary lateral incisors and were treated between 2011 and 2018 were asked to participate in the study. Patients with oligodontia or cleft lip and palate were excluded.

Due to the nature of being a retrospective study, no clinical protocols for implant-, prosthetic-, or orthodontic treatments were established prior to treatment. For the later cohort, the implant installations were made before the patients were 25 years old, which was similar to the EC. All but three of the IT cases had undergone orthodontic treatment. Seven different specialists in oral and maxillofacial surgery performed the surgical procedures, and five different specialists in prosthodontics performed the prosthetic treatment. Thirty-nine patients (18 male and 21 female) who had undergone implant therapy met the inclusion criteria and were contacted by letter and phone to be informed of the study. Six were excluded since they were inaccessible. Twenty-two (12 male and 10 female) patients agreed to participate in the study, implant treatment group (IT group), all of whom were born between the years 1994 and 1998.

The space closure group (SC group) in LC consisted of patients treated at the Department of Orthodontics, Jönköping, Sweden. Fifty-one patients (11 male and 40 female) met the inclusion criteria. Sixteen of these were excluded since they were inaccessible. Twenty-two (two male and 20 female) patients agreed to participate, all of whom born between the years 1990 and 2004. All of the patients in the SC group had been treated with fixed appliances by eight different specialists in orthodontics.

An examination was performed by one of the authors (CH) at least 5 years after insertion of the prosthetic replacements in the IT group and after completed orthodontic treatment in the SC group.

The LC examined in this study (*n* = 44) was compared to a cohort (EC) studied and described in a previous study by Josefsson *et al*. [24]. EC comprised patients (*n* = 44) treated due to missing maxillary lateral incisors. In the EC, 28 teeth were IT cases, and the remaining 34 were SC cases. In the LC, 22 patients had 34 lateral incisors replaced with implant-supported crowns. The remaining 22 patients had 36 lateral incisors replaced by canine substitution. One hundred and thirty-two teeth were analysed, with 62 teeth in the EC and 70 teeth in the LC. Patients examined (LC) were matched to the Josefssons cohort (EC) with respect to the number of patients, diagnosis, and treatment to enable comparisons between the cohorts, hence enabling a comparison of patients treated with a 10-year difference. The study groups are presented in Table 1.

**Table 1.** Presentation of study groups.

Cohort	Treatment	*N*	Age	Sex		Agenesis		Number of teeth	Years since treatment
			(years)	Male	Female	Unilateral	Bilateral		
EC	IT	22	24.6–33.7	8	14	16	6	28	6–13
	SC	22	20.5–30.7	8	14	10	12	34	5–9
	Total	44	20.5–33.7	16	28	26	18	62	5–13
LC	IT	22	23.0–28.0	12	10	10	12	34	5–8
	SC	22	19.5–31.5	2	20	8	14	36	5–9
	Total	44	19.5–31.5	14	30	18	26	70	5–9
EC & LC		88	19.5–33.7	30	58	44	44	132	5–13

Presentation of the early cohort (EC) and latter cohort (LC), and the distribution of cases and teeth between the two treatment methods. Implant treatment (IT) and orthodontic space closure (SC).

The IT and SC groups within each cohort were matched with respect to diagnosis, treatment year, and the number of patients.

The clinical examinations for both cohorts assessed three categories:

The aesthetics of the tooth replacing the missing maxillary lateral incisor:
  a. Crown colour (optimal, acceptable, or not acceptable)
  b. Colour of the adjacent gingiva (normal, acceptable, or not acceptable)
  c. Crown length (normal, short, or long, in relation to the central incisors)
2. The gingival conditions of the tooth replacing the missing maxillary lateral incisor:
  a. Visible implant (yes or no)
  b. Gingival recession, buccally (in mm, measured from the cemento-enamel junction/porcelain edge to the gingival margin to the nearest 0.5mm)
  c. Bleeding on probing (six sites for each tooth)
  d. Papillae formation according to Jemt [25]
    i. Score 0. No papilla present
    ii. Score 1. Less than half of the height of the papilla is present
    iii. Score 2. Half or more of the height of the papilla is present but does not extend all the way up to the contact point between the teeth.
    iv. Score 3. The papilla fill up the entire proximal space and is in good harmony with the adjacent papilla.
    v. Score 4. The papilla is hyperplastic and covers too much of the single-implant restoration and/or adjacent tooth.
  e. Suppuration on probing (yes or no)
3. Occlusal morphology and extra oral assessment:
  a. Sagittal dental relationship (Angle classification I, II or III)
  b. Space conditions (normal, spacing, or crowding)
  c. Overjet (the distance from the most labial point of the incisal edge of the maxillary incisor to the most labial surface of the corresponding mandibular incisor. Measured to the nearest 0.5 mm, parallel to the occlusal plane)
  d. Overbite (measured vertically from the incisal edge of the most inferior maxillary incisor to the incisal edge of the corresponding mandibular incisor. Measured to the nearest 0.5 mm)
  e. Inclination of maxillary incisors (normal, proclined, or retroclined)
  f. Midline in the upper arch (normal or midline shift)
  g. Lip closure (normal or strained)
  h. Overall appearance of dentition when smiling according to specialist (optimal, acceptable, or not acceptable)

Variables within categories one (aesthetics) and two (gingival conditions) were registered for each tooth, whereas variables within category three (occlusal morphology and extra oral assessment) were registered for each patient. Space conditions were assessed anteriorly in the maxilla and noted as spacing when there was excessive space of at least 1 mm and as crowding where the space deficit was at least 1 mm.

In addition to the clinical examination, the patients were asked about their satisfaction of the aesthetic appearance of the anterior teeth. The response was designed with fixed statements: ‘satisfied’, ‘acceptable’, or ‘not acceptable’.

To test the inter-examiner reliability, eight randomly selected participants were examined by the main examiner (CH) and another examiner (EJ). EJ is the examiner responsible for the examinations in the previously performed study used for comparison of data [24]. The variables tested included 1a, 1b, 1c, 2a, 2b, 2d, 3f, 3g, and 3h. The inter-examiner agreement was found to be substantial (0.72 according to weighted Cohen’s Kappa test).

Intra-examiner reliability was tested using photographs taken during the examination. Examiner CH performed a second assessment of 1a, 1b, 1c, 2d, and 3h. This second assessment was performed 1 week after the examination. The intra-examiner agreement was found to be almost perfect (0.89 according to the weighted Cohen’s Kappa test).

### Statistical methods

All statistical analyses were carried out using the statistical software SPSS version 27 (IBM Corp., Armonk, NY, USA). The sample size regarding the number of cases was based on the available sample from the study by Josefsson *et al*. [24]. Their sample size was calculated based on the possibility of detecting a difference of 35% in the rating outcome of crown and gingival appearance, with a significance level of 0.05 and power of 80%. The power analysis showed that 22 cases in each group within each cohort were sufficient. The Shapiro-Wilk test (*P* < .05) and a visual inspection of the histograms, normal Q-Q plots, and box plots of the data showed that the parameters were not normally distributed. The Chi square test was therefore used for the analysis of the nominal data, and the Mann–Whitney *U* test was used for the analysis of the scale data. In the nominal parameters with more than two values, dichotomization was performed.

## Results

### Implant treatment

#### Aesthetics

There were no differences between IT cases when comparing the colour of the gingiva of the two cohorts. In the EC, 17 crowns were of abnormal crown length, compared to 10 in the LC (*P* = .020). All of the substituting teeth were considered to have an acceptable colour (Table 2).

**Table 2.** Aesthetics.

Treatment	Variable	Early cohort (*n* = 62)		Latter cohort (*n* = 70)		*P*
		Number of teeth	%	Number of teeth	%	
IT	Different or non-acceptable gingival colour	17	60.5	25	73.5	NS
	Non-acceptable crown colour	0	0	0	0	NS
	Abnormal crown length (short or long)	17	60.5	10	29.5	.020
SC	Different or non-acceptable gingival colour	3	9	1	3	NS
	Non-acceptable crown colour	7	20.5	0	0	.004
	Abnormal crown length (short or long)	5	14.5	17	47	.005

NS, non-significant.

Comparisons between the early cohort (EC) and latter cohort (LC) with regards of the assessments of the aesthetics of the tooth replacing the missing maxillary lateral incisor. There were a total of 132 teeth with 62 teeth in the EC (28 implant treatment, IT, and 34 space closure, SC) and 70 teeth in the LC (34 IT and 36 SC).

#### Gingival conditions

Bleeding on probing (BoP) adjacent to the implant-supported crowns was noted in 24 teeth in the EC and 18 teeth in the LC, with a significant difference between the two groups (*P* = .007). There was no difference in visible screws, gingival recessions, or suppuration between the IT cases in the different cohorts. When assessing the papillae adjacent to the implant-supported crowns, there were more that were assessed as being defected in the EC compared to the LC (*P* = .024) (Table 3).

**Table 3.** Gingival conditions.

Treatment	Variable	Early cohort (*n* = 62)		Latter cohort (*n* = 70)		*P*
		Number of teeth	%	Number of teeth	%	
IT	Visible implant	1	3.5	4	12	NS
	Buccal gingival retraction	8	28.5	4	11.5	NS
	Bleeding on probing	24	85.5	18	53	.007
	Suppuration	0	0	5	15	NS
	Papillae defect	19	68	13	38	.024
SC	Buccal gingival retraction	5	14.5	4	11	NS
	Bleeding on probing	2	35.5	12	5.5	.002
	Suppuration	0	0	0	0	NS
	Papillae defect	13	38	8	22	NS

NS, non-significant.

Comparisons between the early cohort (EC) and latter cohort (LC), with regards to the gingival conditions of the tooth replacing the missing maxillary lateral incisor. There were a total of 132 teeth with 62 teeth in the EC (28 implant treatment, IT, and 34 space closure, SC) and 70 teeth in the LC (34 IT and 36 SC).

#### Occlusal morphology and extra oral assessment

Angle class I was found in 77% of the IT cases in the EC and 82% in the LC. The overjet varied between 0 and 6 mm (mean = 3 mm) in the EC and 2 and 5 mm (mean = 3.1 mm) in the LC. The overbite varied between −1.5 mm and 7 mm (mean = 3.3 mm) in the EC and 1 and 5 mm (mean = 3 mm) in the LC. There were no significant differences between the cohorts in Angle classifications, overjet, and overbite.

Eleven patients in the EC and nine in LC hade some spacing anteriorly in the maxilla, whereas crowding was rare.

The maxillary incisors in the IT cases were significantly more proclined (*P* < .001), and strained lip closure was more commonly occurring (*P* = .006) in the EC than in the LC. A difference was also found in appearance when smiling between the two cohorts in patients treated with IT. Seven cases were assessed as non-acceptable in the EC, compared to none in the LC (*P* < .001) (Table 4). There were no differences in space conditions, overjet, overbite, or midline when comparing IT cases in the EC and the LC.

**Table 4.** Occlusal morphology and extra oral assessment.

Treatment	Variable	Early cohort (*n* = 22)		Latter cohort (*n* = 22)		*P*
		Number of patients	%	Number of patients	%	
IT	Proclination of maxillary incisors	10	45.5	0	0	<.001
	Strained lip closure	5	22.5	0	0	.006
	Midline deviation maxilla	10	45.5	5	22.5	NS
	Non-acceptable appearance when smiling	7	32	0	0	<.001
SC	Proclination of maxillary incisors	3	13.5	4	18	NS
	Strained lip closure	0	0	1	4.5	NS
	Midline deviation maxilla	9	41	8	36.5	NS
	Non-acceptable appearance when smiling	2	9	0	0	NS

NS, non-significant.

Occlusal morphology and extra oral assessment of the analysed cases treated with implants (IT) and orthodontic space closure (SC) in the two cohorts. Presented based on patients, not on teeth. There were a total of 88 patients, with 44 in each cohort, equally distributed between IT and SC.

#### Patient satisfaction with the aesthetics of the maxillary anterior teeth

As presented by Josefsson *et al*. [24], 15 patients (68%) were satisfied with the aesthetics, six patients (27%) considered the aesthetics to be acceptable, and one of the patients (5%) classified the aesthetics as nonacceptable. In the LC, 17 patients (77%) were satisfied with the aesthetics and five patients (23%) classified the aesthetics as acceptable. No significant difference was found between EC and LC when comparing the IT patient’s assessment of the aesthetics.

### Space closure

#### Aesthetics

There were no differences in the colour of the gingiva between SC cases when comparing the two cohorts. When comparing SC cases in the two cohorts, there was a difference in colour of the crown and length of the crown. More teeth in the EC were considered to have a non-acceptable crown colour than in the LC (*P* = .004), and more of the canines in the LC were considered to have an abnormal length (*P* = .005) (Table 2). In further analyses, it was found that the canines replacing the missing laterals were considered to be too long in the LC (*n* = 15), compared to only one case in the EC (*P* = .009).

#### Gingival conditions

There were no differences in gingival recessions, suppuration, or papillae over time between the cohorts of the SC cases. There were significantly more teeth (*n* = 12) with BoP in the LC than in the EC (two teeth, *P* = .002) (Table 3).

#### Occlusal morphology and extra oral assessment

Angle class I was found in 86% of the SC cases in the EC and 37% in the LC. None of the SC cases were classified as Angle class III. The overjet varied between 1 and 5 mm (mean = 2.8 mm) in the EC and 0 and 4 mm (mean = 2.5 mm) in the LC. The overbite varied between 0 and 7 mm (mean = 3.5 mm) in the EC and 0 and 5 mm (mean = 2.4 mm) in the LC. There were no significant differences between the cohorts in Angle classifications and overjet. There was a difference in overbite when comparing the cohorts, where the EC presented more cases (*n* = 11) with an increased overbite, > 3 mm (*P* = .030).

Fifteen patients in the EC and 11 in the LC had some spacing anteriorly in the maxilla. Crowding was rare, even in the SC cases.

Among the SC cases, there was no difference in any of the other parameters concerning occlusal morphology and extra oral assessment (Table 4).

#### Patient satisfaction with the aesthetics of the maxillary anterior teeth

In the EC, 13 patients (59%) were satisfied with the aesthetics, and nine patients (41%) thought it was acceptable. In the LC, 14 patients (63%) were satisfied with the aesthetics, 7 patients (32%) assessed the aesthetics as acceptable, and 1 patient (5%) considered the aesthetics to be non-acceptable. No significant difference was found between EC and LC when comparing the SC patients’ assessment of the aesthetics.

## Discussion

Two cohorts of cases with missing maxillary lateral incisors ,which were treated within a 10-year interval by either implant treatment or orthodontic space closure, were analysed. The treatments were completed at least 5 years before examination.

When replacing a missing maxillary lateral incisor with the adjacent canine, it is known that the colour of the crown may be a concern and may have a significant effect on the aesthetic outcome [26]. In this study, there were seven teeth assessed as having a non-acceptable crown colour in the EC, while there were none in the LC. This finding may be due to a higher awareness of treatment planning and case selection. None of the SC cases had had any whitening performed.

A difference was found between the cohorts in both IT cases and SC cases when comparing crown length. More of the replacing teeth among the IT cases were assessed as satisfactory in LC compared to EC, while the opposite was found among the SC cases. The canines were assessed as too long when replacing laterals in SC cases. To avoid this, bracket placement and succeeding grinding are of importance [27].

The actual length of the replacing tooth was not measured; it was only assessed and compared to adjacent teeth. There are guidelines for anterior teeth regarding aesthetics: for example, canines and central incisors should be 1–1.5 mm longer than lateral incisors [28]. If the cases in this study had a different height ratio, it was not examined, and if a discrepancy was present, this was not analysed.

A discolouration of the adjacent gingiva in patients treated with implant therapy is a known complication [17, 29]. More than 60% of the implants in the EC and 70% in the LC of the IT cases were considered to have a discoloured adjacent gingiva. Since the patients in the EC were treated, implant materials have developed to allow clinicians to choose more aesthetically favourable materials [22]. In this study, no zirconia abutments or such was used.

A difference was found between the cohorts in BoP in patients treated with implant therapy. Probing force was not calibrated between the two examiners (XX and YY); however, both of the examiners intended to use the recommended 0.25N [30]. Absence of BoP around implants confirms health; however, the presence of BoP may or may not suggest ongoing inflammation [31]. There is an increased risk of traumatic BoP in implants compared to natural teeth, even when light force is used [31].

The difficulties regarding evaluation of the cause of BoP in implants applies to both of the cohorts. The reduced BoP in the LC may, therefore, indicate a healthier gingiva. However, among the implants in the LC, there were five teeth (15%) with suppuration on probing. Suppuration on probing is often associated with peri-implantitis [32]. Peri-implantitis is more prevalent anteriorly [33, 34]. This may be due to factors such as occlusion, thickness of the buccal bone, and thickness of the peri-implant mucosa [35]. Rodrigo *et al*. [34] concluded that as many as 25% of implants anteriorly in the maxilla exhibited peri-implantitis 5 years after installation. In the present study, no radiological examination was performed, hence possible peri-implantitis could not be evaluated [32].

The results of probing around implants are dependent on the profile of the abutments and implants and the shape of the prosthetic construction. The probing may, therefore, result in an underestimation of the extent of the lesion [36]. Probing at implants with the prosthetic construction *in situ* should therefore be interpreted with caution [37]. Probing measurements were therefore not analysed.

Proclination of the maxillary incisors was more common among the IT cases in the EC compared to the LC. In borderline cases where space is to be created to enable implant treatment, there is a risk that the expansion causes proclination of the incisors. A more restrictive approach to IT in treating missing maxillary lateral incisors may favour SC in those borderline cases, hence decreasing the risk of proclined incisors. More cases with strained lip closure was found in EC, which may be related to having proclined incisors. Hence an improvement of the inclination of the incisors and the lip closure in IT cases can be ascertained over time.

The overall appearance when smiling among IT cases was assessed as more pleasing by the examiners in the LC. The positioning of the implant is of utmost importance in determining the aesthetic outcome [38]. A slight deviation of implant position buccally can deteriorate the aesthetic appearance [39]. The improvement in appearance, according to the examiners in this study, may be due to other aesthetic improvements in the surgical procedure, prosthetic treatment, or an improvement in case selection in treatment planning. While an improvement over time was found in IT cases, according to the examiners, no such difference was found in the patients’ assessment of the aesthetics. Nor was there any difference over time between the cohorts in the SC patients’ assessment of the aesthetics.

Some participants contributed with two teeth when analysing variables within categories one (aesthetics) and two (gingival conditions). Initially, all participants contributed with only one tooth, which tooth included in the analysis was decided through randomization. However, since our findings were compared to the previous study by Josefsson *et al*. [24] who included all teeth in their analyse the same was done in this study. Comparisons were made between the randomized analyse and the one including all teeth. This comparison showed no significant difference between the two analyses.

### Limitations

The limited number of analysed cases may have had a negative impact on our ability to identify significant differences.

Two different examiners performed the examinations of the two cohorts. The inter-examiner reliability was adequate, but the use of two different examiners increases the risk of errors in assessments.

There is a difference in years from treatment to examination between the EC and the LC among the IT cases. This difference may have an impact on our findings.

Assessment of the parameters within category one and parameter 3b and 3e are based on a subjective assessment of the authors. Hence, the results mainly reflect the professional assessments.

### Strengths

The time horizon of 10 years enabled the evaluation of the evolution of treatment results in patients missing maxillary lateral incisors.

## Conclusion

Among the IT cases, where a difference was found between the cohorts, an improvement was noted.

When comparing the cohorts concerning space closure, the colour of the crown and overbite had improved over time while the opposite was noted concerning crown length and BoP.

## Data Availability

The data underlying this article will be shared on reasonable request to the corresponding author.
